# Mcl-1 deficiency in murine livers leads to nuclear polyploidisation and mitotic errors: Implications for hepatocellular carcinoma

**DOI:** 10.1016/j.jhepr.2023.100838

**Published:** 2023-07-11

**Authors:** Laure-Alix Clerbaux, Pierre Cordier, Nina Desboeufs, Kristian Unger, Peter Leary, Gabriel Semere, Yannick Boege, Lap Kwan Chan, Chantal Desdouets, Massimo Lopes, Achim Weber

**Affiliations:** 1 Department of Pathology and Molecular Pathology, University Hospital Zürich (USZ), Zurich, Switzerland; 2 Institute of Molecular Cancer Research (IMCR), University of Zürich (UZH), Zurich, Switzerland; 3 Centre de Recherche des Cordeliers, Sorbonne Université, INSERM, Université de Paris, Paris, France; 4 Genomic Instability, Metabolism, Immunity and Liver Tumorigenesis Laboratory, Equipe Labellisée LIGUE 2023, Paris, France; 5 Research Unit Radiation Cytogenetics, Helmholtz Munich, Neuherberg, Germany; 6 Department of Radiation Oncology, University Hospital, LMU Munich, Munich, Germany; 7 Bavarian Cancer Research Center (BZKF), Munich, Germany; 8 Functional Genomics Center Zurich, University of Zürich and ETH Zürich, Zurich, Switzerland

**Keywords:** Liver, Polyploidy, Mcl-1, Chromosome segregation, Mutational signature, Hepatocarcinogenesis

## Abstract

**Background & Aims:**

Mcl-1, an antiapoptotic protein overexpressed in many tumours, including hepatocellular carcinoma (HCC), represents a promising target for cancer treatment. Although Mcl-1 non-apoptotic roles might critically influence the therapeutic potential of Mcl-1 inhibitors, these functions remain poorly understood. We aimed to investigate the effects of hepatic Mcl-1 deficiency (Mcl-1^Δhep^) on hepatocyte ploidy and cell cycle in murine liver *in vivo* and the possible implications on HCC.

**Methods:**

Livers of young Mcl-1^Δhep^ and wild-type (WT) mice were analysed for ploidy profile, mitotic figures, *in situ* chromosome segregation, gene set enrichment analysis and were subjected to two-thirds partial hepatectomy to assess Mcl-1 deficiency effect on cell cycle progression *in vivo*. Mcl-1^Δhep^ tumours in older mice were analysed for ploidy profile, chromosomal instability, and mutational signatures via whole exome sequencing.

**Results:**

In young mice, Mcl-1 deficiency leads to nuclear polyploidy and to high rates of mitotic errors with abnormal spindle figures and chromosome mis-segregation along with a prolonged spindle assembly checkpoint activation signature. Chromosomal instability and altered ploidy profile are observed in Mcl-1^Δhep^ tumours of old mice as well as a characteristic mutational signature of currently unknown aetiology.

**Conclusions:**

Our study suggests novel non-apoptotic effects of Mcl-1 deficiency on nuclear ploidy, mitotic regulation, and chromosomal segregation in hepatocytes *in vivo*. In addition, the Mcl-1 deficiency characteristic mutational signature might reflect mitotic issues. These results are of importance to consider when developing anti-Mcl-1 therapies to treat cancer.

**Impact and implications:**

Although Mcl-1 inhibitors represent promising hepatocellular carcinoma treatment, the still poorly understood non-apoptotic roles of Mcl-1 might compromise their successful clinical application. Our study shows that Mcl-1 deficiency leads to nuclear polyploidy, mitotic errors, and aberrant chromosomal segregation in hepatocytes *in vivo*, whereas hepatocellular tumours spontaneously induced by Mcl-1 deficiency exhibit chromosomal instability and a mutational signature potentially reflecting mitotic issues. These results have potential implications for the development of anti-Mcl-1 therapies to treat hepatocellular carcinoma, especially as hyperproliferative liver is a clinically relevant situation.

## Highlights

• Mcl-1 deficiency leads to mononucleated polyploid hepatocytes accumulation potentially via mitotic slippage.
• Mcl-1 deficiency leads to mitotic spindle assembly checkpoint gene signature and mitotic errors such as aberrant chromosomal segregation.
• Spontaneous hepatocellular tumours induced by Mcl-1 deficiency exhibit chromosomal instability and altered ploidy profile.
• The mutational signature of Mcl-1 deficient liver tumours might reflect mitotic issues.

## Graphical abstract

**Figure:**
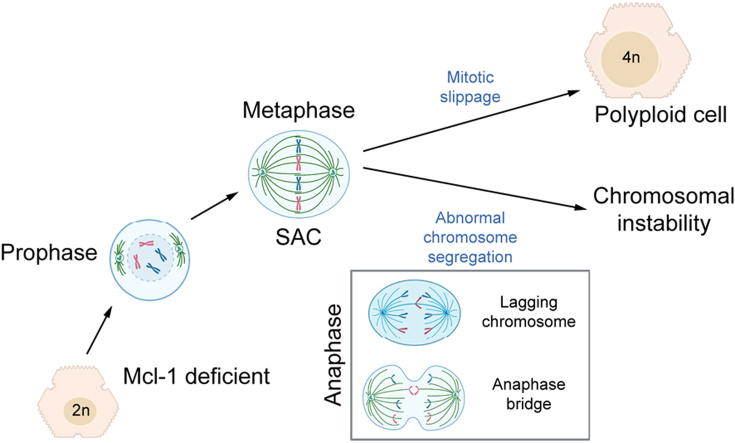

## Introduction

Liver cancer is one of the leading causes of cancer-related death worldwide, and hepatocellular carcinoma (HCC) accounts for the majority of primary liver cancers.^1^^,^^2^ Myeloid cell leukaemia sequence 1 (Mcl-1), an anti-apoptotic Bcl-2 family member, is overexpressed in many tumour types, including HCC.^3^ Mcl-1 overexpression is implicated in cancer drug-resistance, as blocking apoptosis allows cells to survive cytotoxic chemotherapeutic challenge.^3^ Therefore, Mcl-1 inhibitors targeting its pro-survival function are under development.^4^^,^^5^ Counterintuitively, we previously showed that a mouse model with a hepatocyte specific deletion of Mcl-1 (Mcl-1^Δhep^) spontaneously develops HCC with an incidence of 50% at 12 months of age. Persistent apoptosis was associated with proliferation and high apoptotic levels at 2 months correlated with HCC development at 12 months.[6], [7], [8] It was proposed that the proapoptotic environment and concomitant proliferation impose a higher DNA replication rate, increasing the risk of replication-associated DNA damage, leading to genetic instability and tumorigenesis.^8^ This suggests that Mcl-1 deficiency-associated apoptosis has oncogenic potential in the liver but also in the gastrointestinal tract.^9^ Furthermore, in addition to its mitochondrial apoptotic function, Mcl-1 was recently shown in *in vitro* studies to have nuclear roles, such as interacting with proliferating cell nuclear antigen, localising at DNA damage sites, interacting with Ku and promoting homologous recombination-dependent DNA repair following replication stress.^4^^,^[10], [11], [12], [13], [14], [15], [16] The complexity of Mcl-1 regulation and its multiple functions, particularly its poorly understood non-apoptotic roles, represent challenges to the successful clinical application of Mcl-1 inhibitors.^15^^,^^17^ Thus, it is crucial to unravel further the non-apoptotic roles of Mcl-1 in the liver *in vivo*.

Polyploidy characterises up to 90% of adult hepatocytes in mice and around 40% in humans.[18], [19], [20] Polyploid hepatocytes are defined by the number of nuclei per cell (cellular ploidy) as well as the DNA content of each nucleus (nuclear ploidy).^21^ In the liver, physiological polyploidisation starts from weaning and increases with age,^22^ whereas pathological polyploidisation is observed in many chronic liver diseases.^23^ Various mechanisms promote polyploidisation.^24^ Cell fusion occurs during viral infections or through receptor-ligand interactions. Cytokinesis failure results in the genesis of binucleated cells. Endoreplication encompasses endocycling (alternation of G and S phases) and endomitosis with mitotic slippage (abortion of mitosis by skipping metaphase or anaphase),^24^ and thus is a cell cycle-dependent process. Interestingly, Mcl-1 has been shown to interact with various regulators of the cell cycle inducing divergent outcomes.^17^ However, these roles have been studied only *in vitro*. Two-thirds partial hepatectomy (PHx), extensively used in rodents to study liver regeneration in which hepatocytes synchronously re-enter cell cycle, represents a powerful tool to investigate the effect of Mcl-1 deficiency on cell cycle progression *in vivo*.

How ploidy and cell cycle contribute to carcinogenesis is a field of deep investigation. Different defective DNA maintenance mechanisms cause somatic mutations, which generate a characteristic mutational signature.[25], [26], [27] The ‘Catalogue Of Somatic Mutations In Cancer’ (COSMIC) lists many signatures identified across the spectrum of human cancers, some of which could be attributed to precise molecular aetiologies,^28^ while molecular origins of many signatures remain unknown. The genomic alterations driving HCC in the different mouse models should be more systematically compared with those found in human tumours to further link mutational signatures with molecular origins.^29^

The unique properties of the liver regarding ploidy dynamic changes and synchronised proliferative capacity following PHx along with the spontaneous development of HCC upon Mcl-1 deficiency led us to investigate the effects of Mcl-1 deficiency on ploidy and cell cycle in murine livers *in vivo,* exploring possible implications for HCC. In addition, we aimed to characterise the mutational signatures of liver tumours arising from Mcl-1 deficiency and compare them with human signatures to potentially link them with underlying mechanisms.

Here, we show that Mcl-1 deficiency in young mouse liver leads to enrichment of mononuclear polyploid hepatocytes and to high rates of mitotic errors with chromosome instability such as spindle asymmetry and aberrant chromosomal segregation along with a prolonged spindle assembly checkpoint (SAC) activation signature. Mcl-1^Δhep^ tumours in old mice showed a high percentage of chromosomal instability and altered ploidy profile. The mutational signatures of Mcl-1^Δhep^ tumours were consistent among all samples and the dominant signature of currently unknown aetiology could potentially reflect mitotic issues.

## Materials and methods

### Animals

All animal experiments conformed to the relevant regulatory standards and were approved by the Swiss Veterinary Office (134/2014, 217/2012, 63/2011, 03/2015 Zurich, ZH104/19). Animals were maintained under pathogen-free conditions and experiments were performed in accordance with the guidelines of the Swiss Animal Protection Law, Veterinary Office, Canton Zurich. Generation of mice with hepatocyte-specific Mcl-1 knockout (homozygous: Mcl-1^flox/flox^-AlbCre [referred to as Mcl-1^Δhep^] and control littermates: Mcl-1^wt/wt^ [referred to as WT]) was done and genotyped as previously described.^6^ Briefly, a conditional Mcl-1 allele was generated by targeting loxP sites upstream of the ATG start codon and between exons 1 and 2.^30^ The region within the two flow sites is lost and the gene is pasted back together again without this region. Mice of approximately 3 weeks of age were fed with a vitamin E-supplemented diet during 4 weeks (thus up to when the mice reached 2 months old).^8^

### Partial hepatectomy

Two-month-old male mice received food and water *ad libitum* before surgery. Mice were anaesthetised by inhalation of isoflurane (2%). Two-thirds PHx was performed between 9 am and 12 am. Three liver lobes, including the gallbladder, were removed. Before and after surgery, mice were treated with analgesia. Mice were euthanised by CO_2_ inhalation and the regenerating liver was harvested at different time points after PHx.

### Statistical analysis

Statistical analysis was performed using GraphPad Prism software (GraphPad Software, San Diego, CA, USA). Data are presented as mean ± SD or mean ± SEM and were analysed by ANOVA. Analysis of two samples was performed with the Student *t* test. Statistical significance is indicated as follows: ∗∗∗∗*p* <0.0001; ∗∗∗*p* <0.001; ∗∗*p* <0.01; ∗*p* <0.05; and n.s., not significant.

For further details regarding the materials and methods used, please refer to the CTAT table and Supplementary information.

## Results

### Mcl-1 deficiency leads to a lower proportion of binucleated hepatocytes concomitant with a higher proportion of mononuclear polyploid nuclei in livers of young mice

**Fig. 1:**
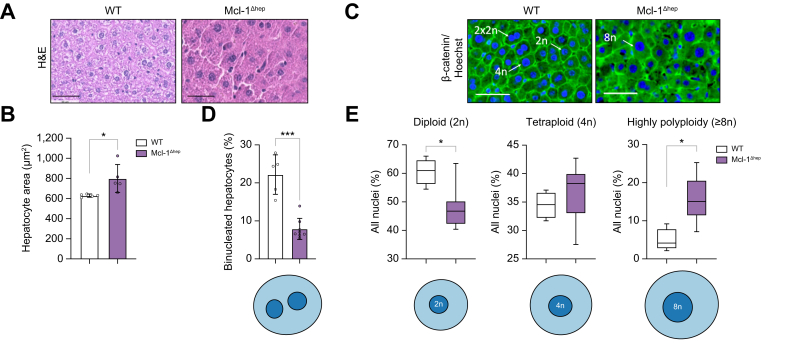
Mcl-1 deficiency leads to a lower proportion of binucleated hepatocytes with a larger proportion of mononuclear polyploidy nuclei in livers of 2-month-old mice. (A) Representative images of H&E staining of livers from 2-month-old Mcl-1^Δhep^ and WT mice. (B) Hepatocyte area. Data represent the mean ± SD (n = 5 per group). (C) Representative images of immunofluorescence labelling of nuclear (Hoechst) and plasma membrane (β-catenin) used to evaluate ploidy. (D) Percentage of binucleated hepatocytes relative to total nuclei in Mcl-1^Δhep^ and WT mice (n = 5 per group). (E) Percentage of mononucleate 2n, 4n, and ≥8n hepatocytes relative to total mononucleate hepatocytes in Mcl-1^Δhep^ and WT mice (n = 4–6 per group). Statistical test: one-way ANOVA test with Tukey’s multiple comparisons test when significant. ∗*p* <0.05; ∗∗∗*p* <0.001. Data are represented as mean ± SEM. H&E, hematoxylin and eosin; Mcl-1, myeloid cell leukaemia sequence 1; WT, wild type.

In Mcl-1^Δhep^ livers, cell density was decreased and hepatocytes exhibited increased cell size (Fig. 1A and B). To determine if cellular ploidy (number of nuclei per hepatocyte) and/or nuclear ploidy (ploidy of each nucleus) were altered, we analysed the hepatocyte ploidy profile using whole-slide imaging approaches by labelling nuclear (Hoechst) and plasma membrane (β-catenin) compartments (Fig. 1C and Fig. S1A). Regarding cellular ploidy, the binuclear fraction was lower in Mcl-1^Δhep^ than in WT livers at 2 months old (Fig. 1D). Regarding nuclear ploidy, the mononuclear diploid (2n) hepatocyte nuclei were substantially lower in Mcl-1^Δhep^ than in WT livers, whereas the proportion of mononuclear tetraploid nuclei was similar (Fig. 1E). Strikingly, Mcl-1^Δhep^ livers were enriched in highly polyploid nuclei (≥8n) (15.9 ± 5.7%) which were less frequently observed in WT livers (4.9 ± 2.8%) (Fig. 1E). The data show that Mcl-1 deficiency leads to a higher proportion of enlarged mononuclear polyploid hepatocytes in livers of young mice.

### Mononuclear polyploid nuclei accumulate in livers deficient for Mcl-1 independently of apoptotic activity, age, or oxidative stress levels

**Fig. 2:**
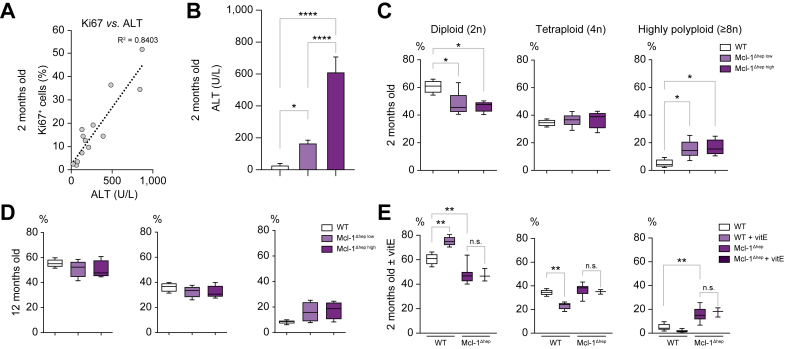
Mononuclear polyploidy nuclei accumulate in hepatocytes lacking Mcl-1 independently of apoptotic activity, age, or oxidative stress in mice. (A) Correlation between serum ALT levels (surrogate marker of apoptosis) and the percentage of Ki67+ hepatocytes (surrogate marker for proliferation) in livers of 2-month-old Mcl-1^Δhep^ mice (n = 13) (R^2^ = 0.84). (B) Serum ALT levels in 2-month-old mice (n = 4–7 per group). (C) Percentage of mononucleate 2n, 4n, and ≥8n hepatocytes relative to total mononucleate hepatocytes in 2-month-old Mcl-1^Δhep^ and WT mice (n = 4–6 per group). (D) Percentage of mononucleate 2n, 4n, and ≥8n hepatocytes relative to total mononucleate hepatocytes in livers of 12-month-old Mcl-1^Δhep^ and WT mice (n = 4–7 per group); and (E) in livers of mice treated or not with vitamin E for 4 weeks (n = 3–7 per group). Statistical test: one-way ANOVA test with Tukey’s multiple comparisons test when significant. n.s., not significant. ∗*p* <0.05, ∗∗*p* ≤0.01, ∗∗∗∗*p* ≤0.0001. Data are expressed as mean ± SEM. ALT, alanine transaminase; Mcl-1, myeloid cell leukaemia 1; WT, wild type.

We previously showed that hepatocyte-specific deletion of Mcl-1 triggers apoptosis and compensative proliferation in livers of 2-month-old mice (Fig. 2A) with higher hepatic apoptotic levels at 2 months correlating with HCC development at 12 months.^8^ To evaluate the possible link between polyploidy and increased apoptosis in our model, we analysed the nuclear ploidy profile in Mcl-1^Δhep^ livers displaying low *vs.* high serum alanine transaminase (ALT) levels. We found no differences in nuclear ploidy as a function of apoptosis level (Fig. 2B). Of note, as in controls, highly polyploid hepatocytes were distributed evenly throughout all the zones of the lobule regardless of ALT levels (Fig. S1B). As 2 months of age might be too early to observe apoptotic-associated differences, we analysed nuclear ploidy in 12-month-old mice, but still observed no ploidy profile differences in Mcl-1^Δhep^ livers between high and low ALT levels at 2 months (Fig. 2C). To exclude that the reduction in 2n nuclei and amplification of 8n nuclei contingent in Mcl-1^Δhep^ livers reflected simply the proliferative activity of Mcl-1^Δhep^ livers, we assessed the percentage of phospho-histone H3 (pHH3)-positive nuclei (marker of cells in G2/M phase) in each nuclear ploidy category. Only a small fraction of 4n and 8n nuclei were pHH3+ in Mcl-1^Δhep^ livers (2% and <8%, respectively), independently of the apoptotic levels, indicating that the apparent tetraploid and octaploid nuclei were not nuclei in G2/M, thus unlikely to reflect increased proliferative activity in Mcl-1^Δhep^ livers at 2 and 12 months (Fig. S2A–C).

Oxidative stress was shown to promote the appearance of highly polyploid cells in fatty liver murine settings, and antioxidant-treated hepatocytes returned to physiological states of ploidy.^31^ As shown previously, Mcl-1^Δhep^ livers of mice on a chow diet exhibited 8-hydroxy-2′-deoxyguanosine (8-OHdG)-positive hepatocytes, indicating oxidative stress, not observed anymore when mice were treated with antioxidant (vitamin E).^8^ Mcl-1^Δhep^ mice of approximately 3 weeks of age treated for 4 weeks with normal or antioxidant diet showed comparable levels of DNA damage as assessed by ɣH2AX (Fig. S2D). In WT livers, antioxidant treatment increased 2n nuclei and decreased 4n nuclei proportion (Fig. 2D). By contrast, Mcl-1^Δhep^ mice treated 4 weeks with this antioxidant diet were enriched in mononuclear polyploid nuclei similarly to untreated Mcl-1^Δhep^ mice (Fig. 2D).

Overall, these data indicate that polyploid hepatocytes accumulate in Mcl-1^Δhep^ livers independently of the apoptotic levels and are not transient G2/M hepatocytes. Although antioxidant treatment altered the ploidy profile of WT livers, the data showed that oxidative stress does not contribute to the accumulation of polyploid hepatocytes in livers deficient in Mcl-1.

### Mcl-1 deficiency leads to prolonged spindle assembly checkpoint activation and high rates of mitotic errors including abnormal chromosomal segregation

**Fig. 3:**
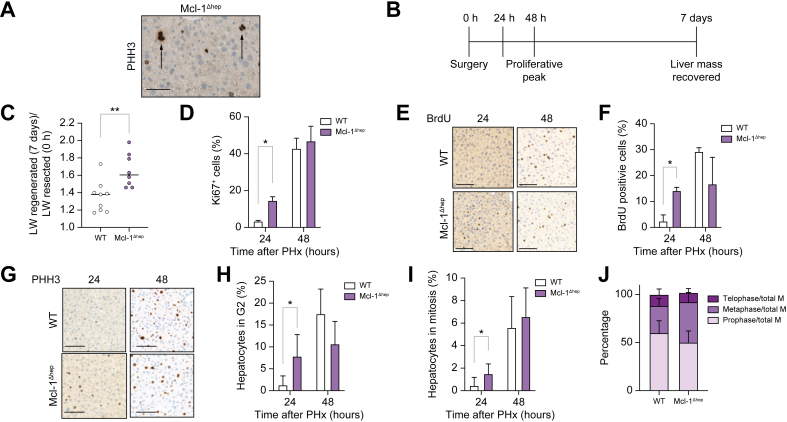
Mcl-1 deficiency impacts progression through cell cycle. (A) Representative image of a liver of a 2-month-old Mcl-1^Δhep^ mouse stained with pHH3, a marker of cells in G2 or mitosis. White arrows show hepatocytes entering mitosis. (B) Timing of experimental procedure for PHx. (C) Ratio of liver weight regenerated (T = 7 days) on liver weight resected (T = 0 h). (D) Percentage of Ki67+ cells (hepatocytes in cell cycle) 24 h and 48 h after PHx. (E, G) Representative BrdU and pHH3 labelling and (F, H, I) quantitative analysis of BrdU (hepatocytes in S phase) and pHH3 labelling (hepatocytes in G2 and mitosis) in livers of 2-month-old Mcl-1^Δhep^ and WT mice 24 h and 48 h after PHx. Data represent mean ± SD (n = 3-6 per group). Statistical test: one-way ANOVA with Tukey’s multiple comparisons test when significant. ∗*p* <0.05; ∗∗*p* ≤0.01. (J) Percentage of prophase, metaphase, and telophase events in WT and Mcl-1 liver tissue sections immunostained with PHH3 48 h after PHx. Telophase events include anaphase ones (n = 4). Data are represented as mean ± SEM. BrdU, bromodeoxyuridine; LW, liver weight; Mcl-1, myeloid cell leukaemia sequence 1; pHH3, phospho-histone H3; PHx, partial hepatectomy; WT, wild type.

**Fig. 4:**
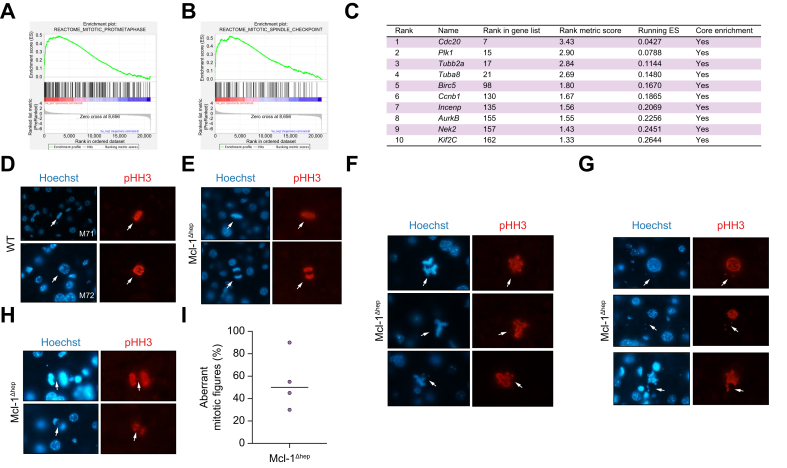
Mcl-1 deficiency leads to prolonged SAC activation, mitotic errors and chromosome mis-segregation in steady-state livers of 2-month-old mice. (A, B) GSEA comparing all differentially regulated genes from livers of 2-month-old Mcl-1^Δhep^ mice with various gene sets. (C) Table of the 10 most enriched genes involved in the regulation of metaphase obtained by GSEA analysis of 2-months-old WT *vs.* Mcl-1^Δhep^ mice. (D). Representative images of Hoechst and pHH3 staining of livers of 2-month-old WT mice displaying normal prophase nucleus and symmetric bipolar metaphase plate. Representative images of Mcl-1^Δhep^ mice displaying (E) normal and (F) abnormal mitotic figures with spindle multipolarity and asymmetry, (G) chromosome exclusion as well as (H) lagging and bridging chromosomes. (I) Percentage of aberrant mitotic figures relative to normal mitotic figures in Mcl-1^Δhep^ livers. GSEA, gene set enrichment analysis; Mcl-1, myeloid cell leukaemia sequence 1; pHH3, phospho-histone H3; SAC, spindle assembly checkpoint; WT, wild type.

We then aimed to investigate which mechanism(s) lead to accumulation of polyploid mononuclear hepatocytes in Mcl-1^Δhep^ liver in young mice. The key event of endocycling is the inhibition of entry into mitosis.^24^ pHH3 staining clearly showed Mcl-1^Δhep^ hepatocytes in mitosis, disfavouring endocycling as the main mechanism leading to nuclear polyploidisation (Fig. 3A). Endomitosis stems for issues arising during mitosis, such as prolonged SAC activation.^24^ We subjected 2-month-old Mcl-1^Δhep^ and WT mice to two-thirds PHx. PHx triggers synchronous hyperproliferation of hepatocytes of the remnant liver with a peak of proliferation at 48 h and a restored liver mass 7 days post-surgery (Fig. 3B). In Mcl-1^Δhep^ livers, the regenerated (7 days) to resected (0 h) liver weight ratio was significantly higher (Fig. 3C and S3A–C). Ki67+ cells appeared already at 24 h post-PHx in Mcl-1^Δhep^ compared with WT (Fig. 3D), whereas Ki67+ cells were virtually absent in both WT and Mcl-1^Δhep^ livers at 6 h post-PHx (Fig. S3D) suggesting that the basal proliferation in Mcl-1^Δhep^ is not the main reason for faster regeneration. When assessing hepatocyte replication by monitoring bromodeoxyuridine (BrdU) incorporation, BrdU was detected already after 24 h in Mcl-1^Δhep^ livers and peaked in both WT and Mcl-1^Δhep^ mice 48 h post-PHx (Fig. 3E and F). Consistently, pHH3 labelling (G2/M marker) showed a similar mitotic index at 48 h post-PHx (Fig. 3G–I). However, in contrast to WT, some Mcl-1-deficient hepatocytes accumulated in the G2/M phase already at 24 h post-PHx. Moreover, although the mitotic index was similar (Fig. 3I) at 48 h post-PHx, the metaphase-to-telophase ratio had a tendency to be higher in Mcl-1^Δhep^ hepatocytes (Fig. 3J). Problems in metaphase–telophase transition have been linked to endomitosis, in which prolonged SAC activation in metaphase bypasses mitosis, known as mitotic slippage. Gene set enrichment analysis (GSEA) in steady-state livers of 2-month-old mice showed an association between genes upregulated upon Mcl-1 deletion and a mitotic SAC gene signature as well as a prometaphase signature (Fig. 4A and B). Transcriptomic analysis from those livers revealed that among the most differentially expressed genes in Mcl-1^Δhep^ livers were *Cdc20*, required for microtubule process, the mitotic kinases *Plk1*, Aurora kinase A and B as well as *Kif2c*, encoding a microtubule protein kinesin (Fig. 4C), that is, all genes involved in mitotic regulation. We thus investigated the formation and polarisation of the microtubule mitotic spindles by assessing the morphology of mitotic figures in Mcl-1^Δhep^ hepatocytes in livers of 2-month-old mice using pHH3 staining. Expectedly, mitotic figures were rare in WT livers but all exhibited normal, symmetric, and bipolar metaphase plates or normal prophase nucleus (Fig. 4D). By contrast, in Mcl-1^Δhep^ livers, in addition to normal mitoses showing symmetrical and bipolar metaphase plate and anaphase (Fig. 4E), aberrant mitotic figures were frequently observed, including spindle multipolarity, spindle asymmetry, and abnormal spindle geometry with metaphase plate asymmetry (Fig. 4F). SAC ensures that chromosomes segregate correctly during cell division.^32^ Upon spindle damage, cells become transiently arrested at the metaphase–telophase transition and can exit mitosis without proper segregation of sister chromatids. Abnormal chromosome position, such as anaphase bridging and chromosome lagging (Fig. 4G), as well as prophase and prometaphase nuclei with chromosome exclusion (Fig. 4H) were observed in hepatocytes lacking Mcl-1. Quantitative β-tubulin/DAPI staining showed that approximately half of the mitotic events were aberrant upon Mcl-1 deletion (Fig. 4I).

Those findings support that, in young mice, hepatocytes lacking Mcl-1 do not undergo endocycling, enter faster into mitosis, show a SAC overactivation signature, and exhibit high rates of mitotic errors with abnormal spindle figures and chromosome mis-segregation.

### Chromosomal instability and characteristic mutational signatures are observed in Mcl-1^Δhep^ tumours

**Fig. 5:**
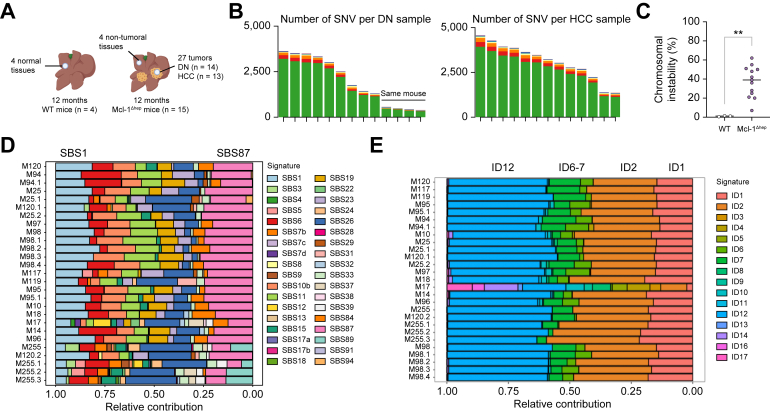
Chromosomal instability, heterogeneous genetic mutations but consistent mutational signatures are observed in Mcl-1^Δhep^ tumours of 12-month-old mice. (A) WES was performed on DNA isolated from 25 discrete neoplasms excised from the livers of 15 Mcl-1^Δhep^ mice. As controls, DNA from four non-tumoral parts of Mcl-1^Δhep^ livers and four WT livers was isolated. (B) Number of SNV deletions in the coding regions per sample of Mcl-1^Δhep^ DN and HCC (n = 13). (C) Percentage of the mean SNV and indel derived chromosomal instability in normal tissues of WT mice (n = 4) and in tumoral tissues of Mcl-1^Δhep^ HCC (n = 13). ∗∗*p* ≤0.01. (D, E) SBSs and small ID COSMIC signatures of Mcl-1^Δhep^ DN and HCC samples (n = 26). DN, dyplastic nodule; COSMIC, catalogue of somatic mutations in cancer; HCC, hepatocellular carcinoma; ID, insertion and deletion; Mcl-1, myeloid cell leukaemia 1; SBS, single-base substitution; SNV, single nucleotide variation; WES, whole exome sequencing; WT, wild type.

Errors in chromosome segregation lead to structural and numerical chromosomal abnormalities, called chromosomal instability (CIN). To assess the extent of CIN upon Mcl-1 deficiency, whole exome sequencing (WES) was performed on DNA isolated from 27 discrete hepatocellular neoplasms excised from the livers of a total of 15 Mcl-1^Δhep^ mice, which were histologically classified as dysplastic nodules (DNs, n = 14) or as HCC (n = 13). As controls, DNA from four non-tumoral parts of Mcl-1^Δhep^ livers and four WT livers was isolated (Fig. 5A). Sequencing data were processed to identify single nucleotide variations (SNVs) with substitutions, small insertions and deletions (indels) in the coding regions. The number of SNVs was high in Mcl-1^Δhep^ DN (except for one mouse for which four DNs were analysed) and in HCC (Fig. 5B). The heatmap of the top 50 genes with differential variant frequencies showed coherently a stronger SNV burden in HCC compared with DN (Fig. S4A). Missense mutations were prevalent with C>T substitutions mainly found in the exomes of Mcl-1^Δhep^ tumours as the samples were paraffin embedded (Fig. S4A). The mean SNV and indel derived CIN was 33.8% (range: 9.1–67.7%) in the Mcl-1^Δhep^ tumours compared with <1% in normal tissues (Fig. 5C).

We then characterised the mutational signatures of tumours arising from Mcl-1 deficiency to compare them with human COSMIC signatures, and where possible, to assign them to known molecular aetiologies. As the formalin-fixed paraffin-embedded (FFPE)-signature is highly similar to signature 30, observed in all our samples, we introduced *FFPEsig*, an algorithm recently developed to rectify the formalin-induced artefacts in the mutational catalogue.^33^ Following this correction, the single-base substitutions (SBSs) COSMIC signatures which most accurately reconstructed the mutational profile of Mcl-1^Δhep^ tumours were SBS1 and SBS87, as well as SBS6, SBS11, and SBS26 in both DN and HCC (Fig. 5D). Small insertion-and-deletion (ID) signatures ID1 and ID2 were found in 20–38% of relative contribution in DN and HCC (Fig. 5E). These mutations correlate with aging and are found in almost all cancers.^26^ ID1 and ID2 indels usually correlate with the numbers of SBS1 substitutions, and are proposed to reflect the number of mitoses a cell has experienced. ID12, the predominant signature, found in all tumours with around 45% of relative contribution, is of unknown aetiology.

Overall, the data showed that Mcl-1^Δhep^ liver tumours display chromosomal instability and a characteristic mutational signature of unknown aetiology.

### Ploidy profile is altered in Mcl-1^Δhep^ tumours

The role of highly polyploid hepatocytes in HCC is still unclear. Binuclear ploidy was strongly reduced in Mcl-1^Δhep^ livers, both in non-tumoral or tumoral tissues, compared with WT livers (Fig. S5A). The nuclear ploidy profile of Mcl-1^Δhep^ tumours exhibited an increase of 4n nuclei compared with Mcl-1^Δhep^ non-tumoral tissues (Fig. S4B). Only a very small fraction of 4n nuclei were pHH3+ in Mcl-1^Δhep^ livers, indicating that proliferation is unlikely to account for the observed increased 4n population in Mcl-1^Δhep^ tumoral hepatic tissues (Fig. S4C). Thus, the ploidy profile is altered in liver tumours spontaneously developing upon Mcl-1 deficiency with reduced cellular ploidy and increased 4n contingent.

## Discussion

Mcl-1 is unique within the Bcl-2 family with respect to its short half-life and acts as a rapid sensor regulating cell death and other important cellular processes.^4^^,^^15^^,^^17^^,^^34^ Many studies have investigated the multiple functions and complex regulation of Mcl-1. However, its non-apoptotic roles await *in vivo* characterisation to develop successful Mcl-1 inhibitors for cancer therapy.

### Effects of Mcl-1 deficiency on nuclear ploidy in the murine liver *in vivo*

Our study shows that Mcl-1 influences hepatocyte ploidy. Mcl-1 deficiency leads to less binucleated and more mononucleated polyploid contingent in the livers of young mice. This is consistent with a previous observation of enlarged cells and reduced binucleation upon Mcl-1 loss obtained with a different knockout strategy^35^ owing to unknown mechanisms. Here, we demonstrated that polyploid nuclei accumulate in Mcl-1 deficient livers independently of apoptotic and proliferative levels. Accumulation of polyploid nuclei has been observed in other chronic liver disease mouse models and was attributed for fatty liver diseases to oxidative stress and associated DNA damage blocking G2/M.^30^^,^^36^ Here we showed that polyploid nuclei accumulate independently of oxidative stress levels in liver lacking Mcl-1 as antioxidant treatment did not rescue the nuclear profile in those livers. In addition, we found an overactivated SAC signature in livers deficient for Mcl-1. Cells that cannot satisfy the SAC are delayed in mitosis^37^ and then may bypass metaphase. No differences were observed in the mitotic index 48 h post-PHx but the metaphase-to-telophase ratio had a tendency to be higher upon Mcl-1 loss. We can hypothesise that some hepatocytes lacking Mcl-1 undergo endomitosis via mitotic slippage inducing nuclear polyploidy in the next G1 phase. A slow degradation of cyclin B with simultaneously active SAC and inactive anaphase promoting complex (APC/C) complex is required for mitotic slippage to occur in humans.^38^ Further studies are required to evaluate the role of these mitotic actors in an Mcl-1-deficient hepatic setting.

### Effects of Mcl-1 deficiency on cell cycle progression in the murine liver *in vivo*

In untreated cells *in vitro*, Mcl-1 protein is cell-cycle regulated with a peak in S-G2 phases both in nuclei and mitochondria.^13^^,^^39^ Upon mild DNA damage, Mcl-1 is rapidly upregulated (in both mitochondria and nucleus) to antagonise apoptosis during the arrested cell cycle to provide time for DNA damage repair.^10^^,^^12^^,^^13^ What is the impact of Mcl-1 deficiency on the cell cycle? Studies *in vitro* showed no impact on cell cycle progression upon Mcl-1 deletion or inhibition in untreated cells; however, Mcl-1-deficient cells were blocked in G2 after irradiation^11^^,^^39^ and did not show Chk1 phosphorylation and no proper G2/M checkpoint allowing correct DNA repair in response to DNA damage upon genotoxic stress.[11], [12], [13] Thus Mcl-1 deficiency seems to have a different impact on cell cycle in a steady-state context *vs.* upon DNA damage. Interestingly, untreated Mcl-1-deficient cells displayed a basal comet tail (indicative of unresolved DNA damage) that was higher than control cells, suggesting that Mcl-1 could have a role in the repair of both induced and spontaneous DNA damage occurring during normal DNA replication.^11^ The failure to undergo complete mitosis after DNA damage coupled to defective checkpoints, called mitotic catastrophe,^40^ has also been shown to lead to polyploidy with DNA damage.^41^ Here, we observed that Mcl-1^Δhep^ hepatocytes enter faster into S, G2, and M phases *in vivo* but accumulate similarly 48 h post-PHx. Chk1 phosphorylation (serine317) seemed to be not altered upon Mcl-1 deficiency 48 h post-PHx (data not shown). This is intriguing as we previously showed that replicating hepatocytes (48 h post-PHx) exhibit double-strand breaks^8^ which should activate G2/M checkpoint via Chk1 phosphorylation. It would be potentially very informative to further study expression and activity of the DNA damage response actors upon hyperproliferation comparing WT and Mcl-1^Δhep^ hepatocytes. Hyperproliferative liver is a clinically relevant situation, not only after liver resection in patients with HCC. Hence further studies are needed to understand the effect of Mcl-1 loss in this context, which may be conductive to successful clinical application of Mcl-1 inhibitors.

### Effects of Mcl-1 deficiency on chromosomal segregation in the murine liver *in vivo*

Our findings show that Mcl-1 influences chromosomal segregation during mitosis. We showed that Mcl-1 deficiency leads to numerous mitotic errors morphologically identified as aberrant mitotic figures, including multipolar mitosis (with more than two spindle poles) and asymmetrical bipolar mitosis with metaphase plate asymmetry and/or abnormal spindle geometry. This large proportion of mitotic errors is consistent with the numerous chromosomal deletions and amplifications revealed by array-comparative genomic hybridization analysis of Mcl-1^Δhep^ livers.^7^ The transcriptomic signature of overactive SAC identified in steady-state livers of 2-month-old Mcl-1^Δhep^ mice indicate that those mitotic aberrations are detected and triggered mitotic checkpoint complex (MCC) assembly. Unattached kinetochores and incorrect alignment of chromosomes on the metaphase plate prompt SAC activation and cell cycle arrest in metaphase, thereby preventing the chromosomal mis-segregation. However, we found that 2-month-old Mcl-1^Δhep^ livers displayed abnormal anaphases with lagging chromosomes, bridging chromatin (anaphase bridges) and prophase nuclei with chromosome exclusion. Mitotic asymmetry and abnormal spindle geometry during metaphase in Mcl-1^Δhep^ livers might induce abnormal chromosome segregation during anaphase, suggesting that Mcl-1 could be an element of the MCC. Some metaphase mitotic defects trigger less SAC activation. For instance, merotelic attachments of chromosomes, in which a single kinetochore is bound to microtubules emanating from opposite spindle poles, are poorly detected by SAC and may not be corrected, leading to lagging chromosomes during anaphase as observed here.^42^ Importantly, merotely is observed at high frequency during polyploid cell division because of the presence of supernumerary centrosomes and is increasingly recognised as an important mechanism contributing to CIN in polyploid cells.^43^ Further studies are required to clarify whether Mcl-1 has a direct role in sensing/correcting mitotic errors and/or in triggering SAC activation.

### Mcl-1 deficiency leading to nuclear polyploidy and mitotic errors: implications for HCC

The functional role of polyploidy in the liver is still poorly understood, especially regarding implication in HCC development. Many HCC mouse models have been studied in terms of their ploidy dynamics.^30^^,^[44], [45], [46], [47], [48] The ploidy fates seem to depend on the injury and consequent mutational context. High polyploid status seems protective against carcinogenesis except when the p53 pathway is disrupted, in which case it becomes promotive toward oncogenesis.^24^ A study showed that the diploid compared with polyploid state is more susceptible to tumour suppressor loss but similarly susceptible to MYC oncogene activation, indicating that polyploidy differently protects the liver from distinct genomic aberrations.^49^ In humans, mononuclear ploidy spectrum varies between HCC with different molecular features.^20^ In Mcl-1^Δhep^ tumours, *Hras* and *Kras* proto-oncogenes were not mutated and *Ctnnb1, Apc, Braf, Egfr*, and *Pten* were not frequently mutated (Fig. S4A). *Brca2* was mutated in the five tumours arising from the same liver but not in the other livers. Thus Mcl-1^Δhep^ neoplasms did not carry recurrent mutations in specific genes. This is coherent with previous data showing that Mcl-1^Δhep^ tumours are heterogeneous with regard to morphology and immunohistochemistry and that p53 pathway is not a key player in this model.^7^ The homologous recombination deficiency (HRD) score was between 2 and 21, with only three tumours above 10 (data not shown) not favouring HRD as the main mechanism underlying HCC in Mcl-1^Δhep^ livers and further confirming the molecular heterogeneity of the Mcl-1^Δhep^ HCC model.

Two studies also showed that polyploid hepatocytes can divide without mitotic errors or chromosome mis-segregation upon chronic liver damage.^45^^,^^50^ The mitotic defects observed here could represent the consequences of cumulative DNA replication stress from the previous S phase impairing spindle organisation. Under-replicated DNA attributable to replication stress persisting in mitosis have been shown to hamper chromosome segregation.^51^^,^^52^ Cells entering mitosis with under-replicated DNA activate a repair mechanism known as mitotic DNA synthesis (MiDAS). During mitosis, tightly regulated pathways operate to limit the deleterious consequences of replicative stress,^53^ to prevent structural and numerical chromosomal aberrations. Interestingly, although Mcl-1^Δhep^ tumours are heterogeneous at multiple levels, the dominant ID mutational signature found in all the 27 lesions analysed (from a total of 15 livers), is currently of unknown aetiology. As MiDAS is mechanistically and genetically similar to break-induced replication, we can hypothesise that specific mutagenic events are associated with it and might be increased during chronic proliferation as in Mcl-1^Δhep^ livers. MiDAS contribution to genomic mutations is still not yet understood; specifically it is not clear which SBS or ID signatures might be associated. Hence it would be potentially insightful to evaluate if Mcl-1 mutational signatures within liver tumours are linked with mitotic issues, by taking advantage notably of MiDAS sequencing approaches recently performed on the human genome.^54^

In conclusion, our study identified previously undescribed non-apoptotic effects of Mcl-1 deficiency on nuclear ploidy, mitosis regulation, and chromosomal segregation in adult mouse hepatocytes *in vivo*. In addition, the particular mutational signature of Mcl-1^Δhep^ hepatocellular tumours might reflect mitotic issues and deserves further investigation. Although it is not yet determined whether the effects of Mcl-1 deficiency are directly attributable to mitotic regulation or are indirect, that is, secondary to increased replication stress in a hyperproliferative environment, our results have potentially important implications for the development of Mcl-1 inhibitors as HCC therapeutics.

## Financial support

This study was supported by a grant from the 10.13039/100000001Swiss National Science Foundation (SNF 320030_182764) to AW and by the Comprehensive Cancer Center Zurich (CCCZ) Funding Program and the ISREC foundation (Fondation de soutien à la recherche sur le cancer en Suisse) to LAC and ND. PC is a recipient of Plan Cancer INSERM (programme «Soutien pour la formation à la recherche fondamentale et translationnelle en cancérologie») and Ligue Nationale contre le Cancer.

## Conflicts of interest

The authors declare that they have no conflict of interest to disclose in relation to this work.

Please refer to the accompanying ICMJE disclosure forms for further details.

## Authors’ contributions

Designed the study and coordinated experiments: LAC, ML, AW. Performed the *in vivo* and *in vitro* experiments and analysis: LAC, ND, GS. Performed the ploidy analysis: PC, CD. Performed the WES bioinformatic analysis: KU, PL. Performed the GSEA analysis: YB, LKC. Wrote the manuscript: LAC with input from all co-authors.

## Data availability statement

The microarray dataset can be found as E-GEOD-75730. Other raw data that support the findings in this study can be found within this article and are available from the corresponding author upon reasonable request.
