# Supplementary material for: Mcl-1 deficiency in murine livers leads to nuclear polyploidisation and mitotic errors: Implications for hepatocellular carcinoma
Source: JHEP Rep. 2023 Jul 11;5(10):100838. doi: 10.1016/j.jhepr.2023.100838 (PMC10472239; doi:10.1016/j.jhepr.2023.100838)
Supplement: Multimedia component 1 [file mmc1.pdf]

Supplementary materials

Mcl-1 deficiency in murine livers leads to nuclear polyploidisation and mitotic errors: Implications for hepatocellular carcinoma

Laure-Alix Clerbaux, Pierre Cordier, Nina Desboeufs, Kristian Unger, Peter Leary Gabriel Semere, Yannick Boege, Lap Kwan Chan, Chantal Desdouets, Massimo Lopes, Achim Weber.

Table of Contents

Supplemental Figures. 2
Supplemental Methods. 7
Supplemental References. 11

Supplemental Figures

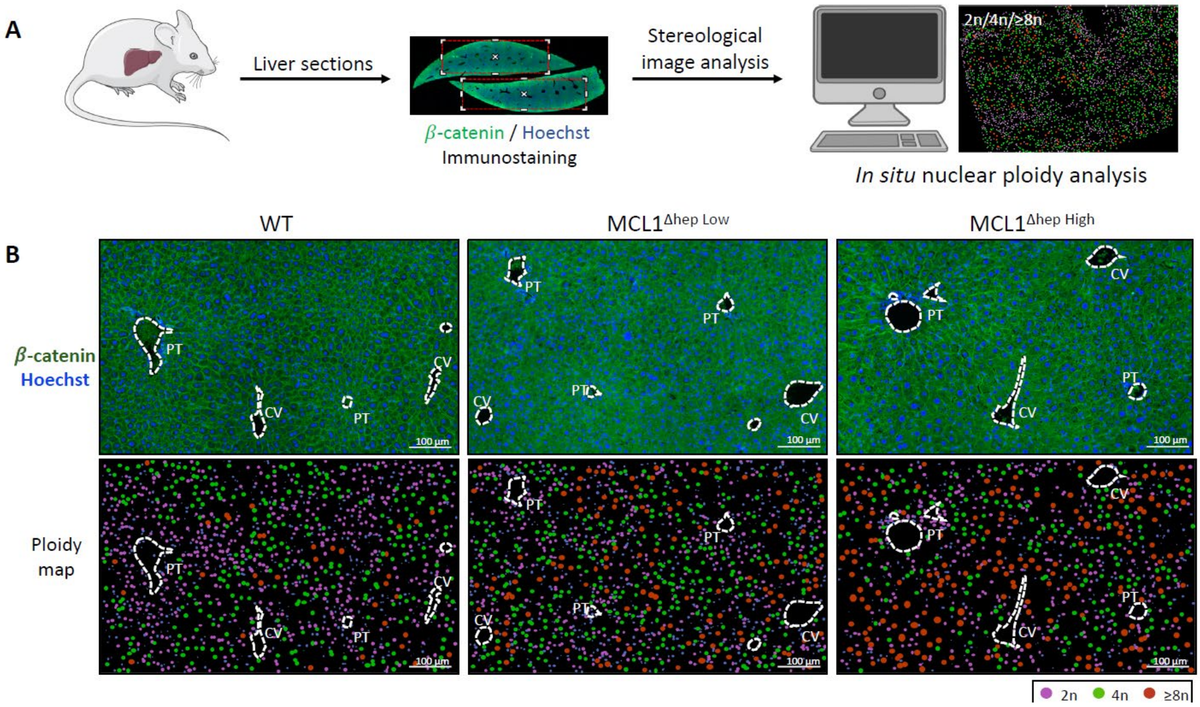

Fig. S1. A. Schematic of the in situ nuclear ploidy analysis. **B.** No specific zonal distribution of the different ploidy contingent was observed between WT mice, Mcl-1$^{\Delta hep}$ mice displaying low or high ALT levels. PT: portal tract; CV: central vein. Blue: Hoechst, green: ß-catenin.

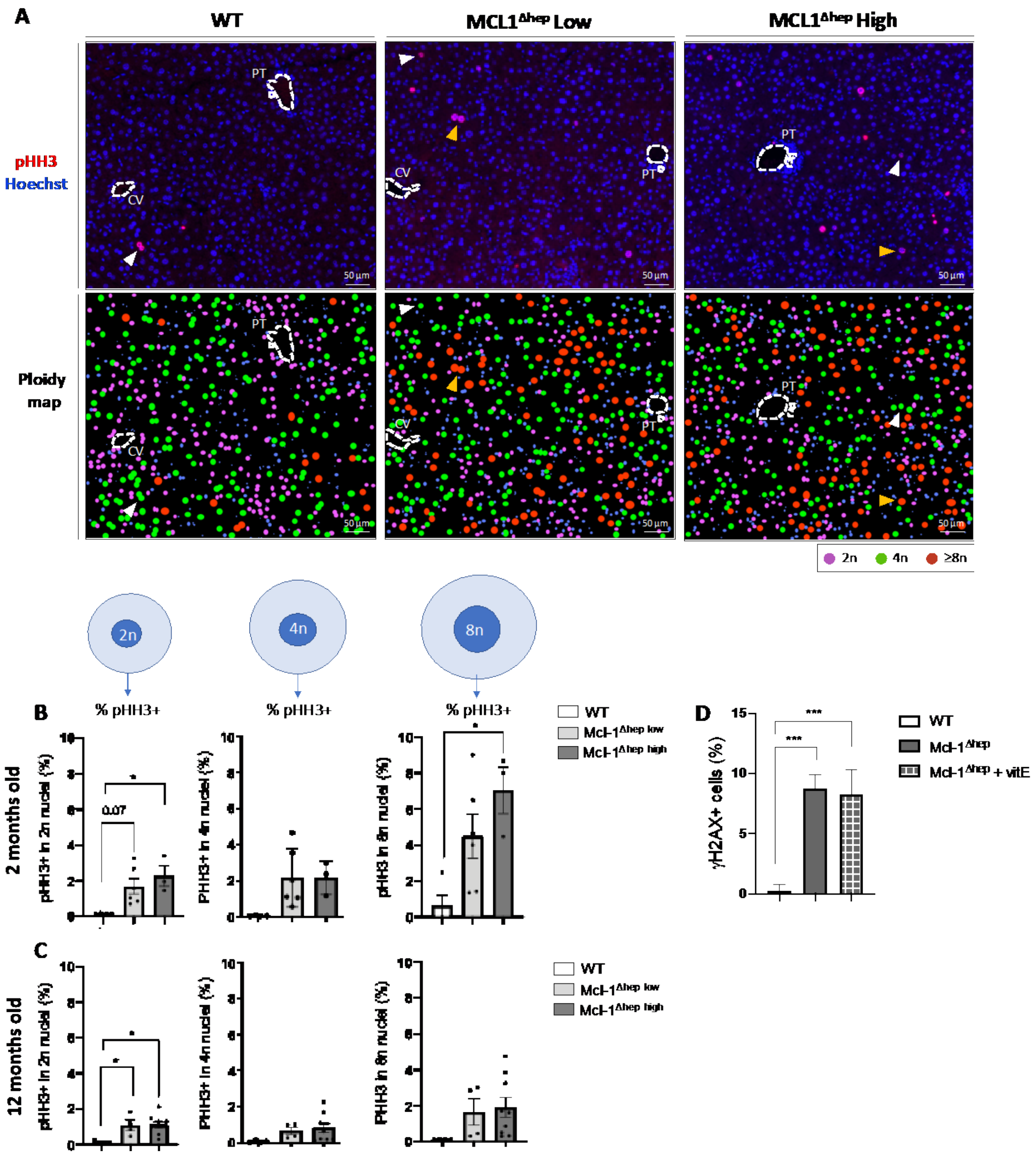

Fig. S2. Mononuclear polyploidy nuclei accumulating in livers lacking Mcl-1 are not transient G2 nuclei. **A**. Representative images of pHH3/Hoechst staining and ploidy map showing the 2n, 4n and 8n nuclear contingents. **B,C.** Percentage of pHH3 positive hepatocytes in mononucleated 2n, 4n and 8n contingent in WT, Mcl-1$^{\Delta hep}$ displaying low or high ATL levels of 2 and 12 months old mice (n=4-8 per group). **D.** Percentage of γH2AX positive hepatocytes in livers of 2 months old WT and Mcl-1$^{\Delta hep}$ mice treated or not with vitamin E for 4 weeks (n=4-6 per group). Statistical test: one-way ANOVA test with Tukey's multiple comparisons test when significant. n.s.: not significant. *p < 0.05, ***p-value ≤ 0.001. Data are expressed as mean ± SEM.

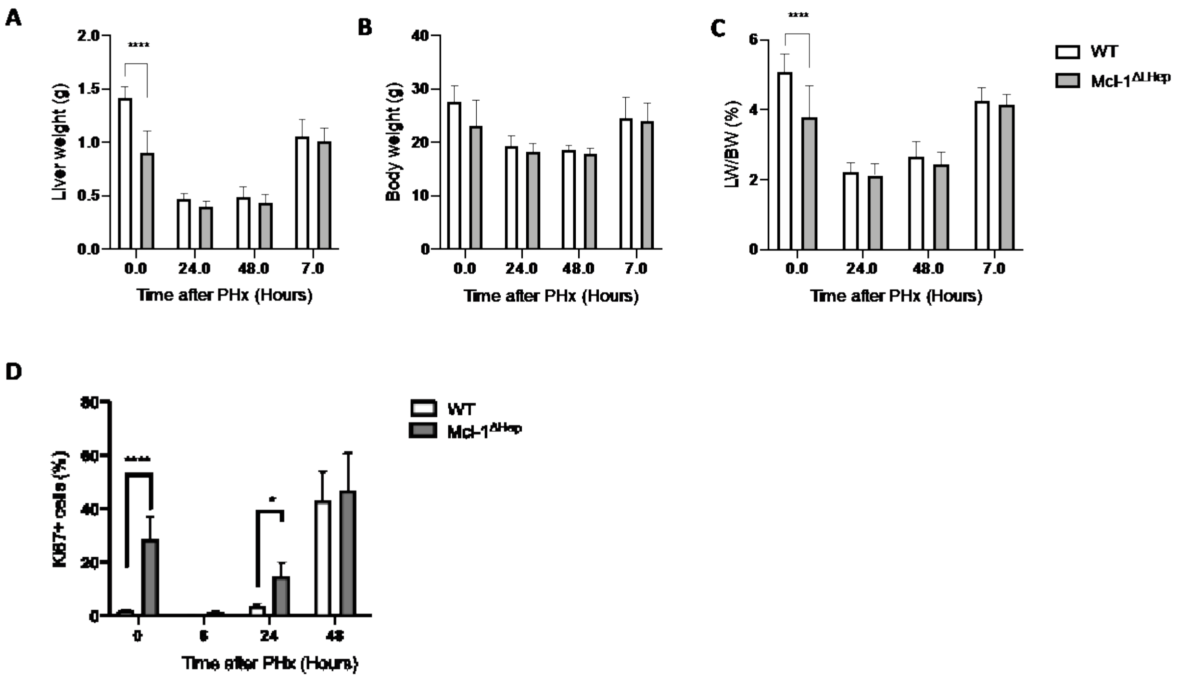

Fig. S3. *Mcl-1 deficiency leads to faster entry into cell cycle.* **A.** Liver weight (LW), **B.** body weight (BW) and **C.** LW/BW ratio at different time points after PHx (n=5-8 per group). **D.** Percentage of Ki67+ cells (hepatocytes in cell cycle) pre-PHx, 6h, 24h and 48h after PHx. (n=4-6 mice per group). Data represent mean +- SD. *$p < 0.05$, ****p-value ≤ 0.0001.

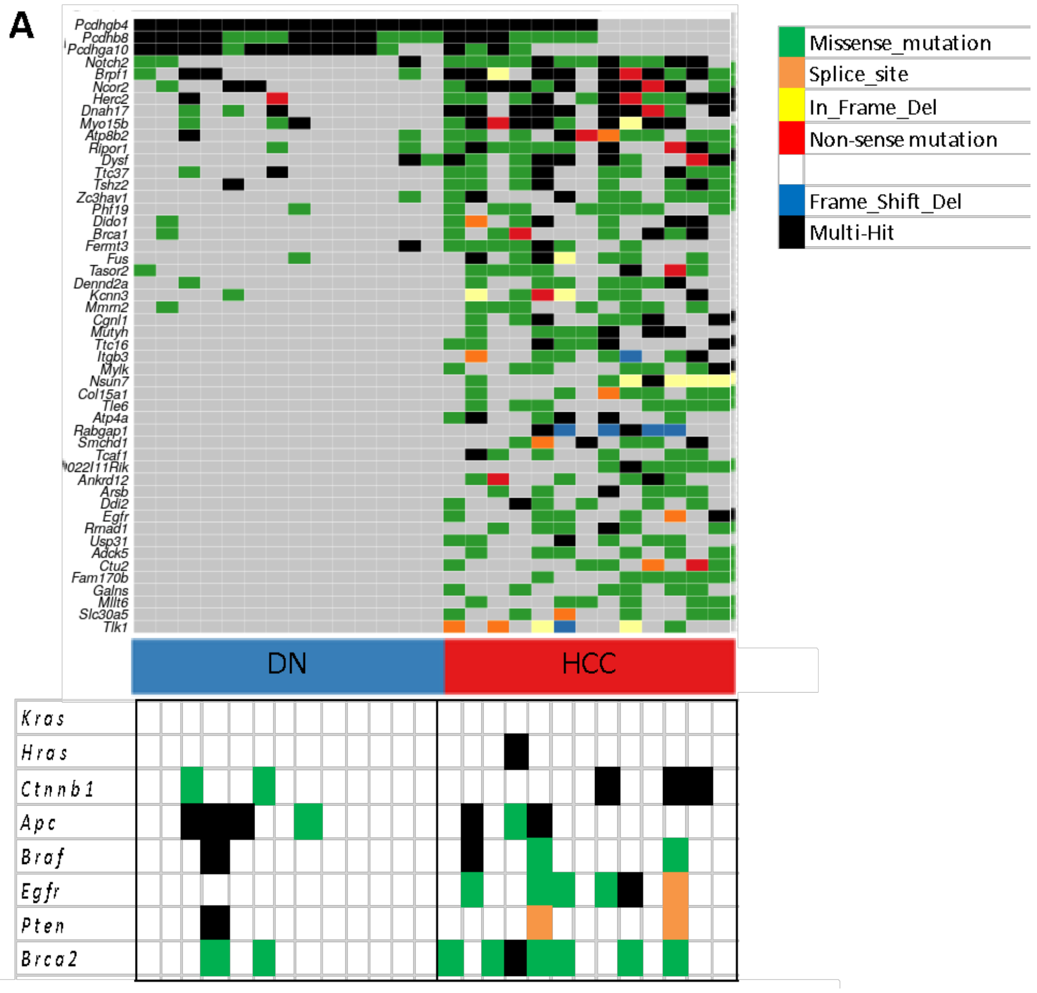

Fig. S4. *Not recurrently mutated genes are observed in Mcl-1$^{\Delta hep}$ tumors of 12 months old mice.* **A.** Plot of the 50 differential genes in DN and HCC Mcl-1$^{\Delta hep}$ samples and mutations in common oncogenes per individual tumor.

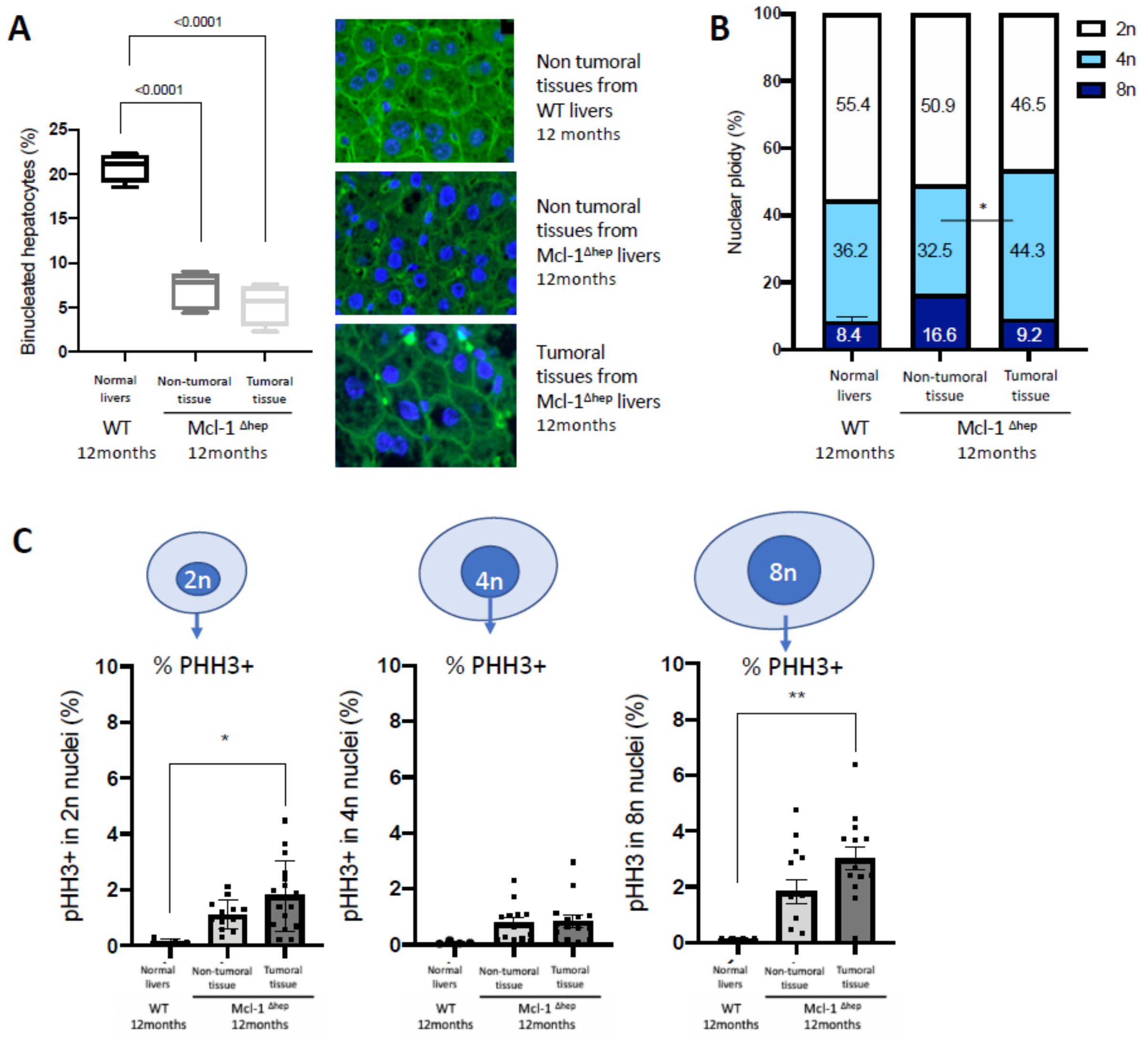

Fig. S5. *Ploidy profile is altered in Mcl-1$^{\Delta hep}$ tumors of 12 months old mice.* **A.** Percentage of binucleated hepatocytes relative to total nuclei in Mcl-1$^{\Delta hep}$ and WT mice (n=4-5 per group) with representative images. **B.** Percentage of mononucleate 2n, 4n and >=8n hepatocytes relative to total mononucleate hepatocytes in livers of 12 months old WT and Mcl-1$^{\Delta hep}$ (n=4 for WT, n=13 for Mcl-1$^{\Delta hep}$). **C**. Percentage of pHH3 positive hepatocytes in 2n, 4n and 8n contingent in normal livers of WT mice, in non-tumoral and tumoral tissues of Mcl-1$^{\Delta hep}$ mice (n=4-10 per group). Data are presented as mean +- SEM. *p<0.05; **p-value ≤ 0.01.

Supplemental Methods

BrdU Assay

For 5-bromo-20-deoxyuridine (BrdU) labelling, BrdU (Sigma-Aldrich, Switzerland; 50 mg/g body weight) was injected i.p. one hour before mice were sacrificed. Detection of BrdU-positive cells was performed by immunofluorescence staining using a peroxidase-coupled antibody against BrdU (1:30; Roche, Switzerland).

Measurement of Serum Parameters

The analysis of aminotransferases ALT was performed in mouse serum with a Roche Modular System (Roche Diagnostics) with a commercially available automated colorimetric system.

Histology and immunostainings

Collected liver tissues were fixed in 10% neutral-buffered formalin, embedded in paraffin, sectioned (5 µm), and stained with Hematoxylin & Eosin (H&E) or specific antibodies for immunohistochemistry or immunofluorescence.

Immunofluorescence staining for hepatocyte ploidy and pHH3 expression analyses was performed manually on 5-µm-thick formalin-fixed paraffin-embedded liver tissue sections. After dewaxing in xylene and rehydration in decreasing serial concentrations of ethanol and distilled H_2O, heat-induced epitope retrieval was performed in tris-based unmasking buffer (VECTOR H3301) (pH 9.0) for 30 minutes in a water bath set at 95°C. Non-specific sites were subsequently blocked with 10% goat serum (Vector S-1000-20) supplemented with 0.1% Triton X-100 (Biorad 161-0407) (permeabilization) diluted in a commercial antibody diluent (ZYTOMED #ZUC025-500) for 1h at room temperature. Sections were incubated with mouse monoclonal anti-β-catenin (BD BIOSCIENCES #610154) or rabbit polyclonal anti-pHH3 (MERCK Millipore #06-570) primary antibody diluted 1:200 in the commercial antibody diluent. After overnight incubation at 4°C, sections were washed in 0.1% Tween 20 Tris-Buffered Saline (TBST1X) and then incubated with Alexa Fluor 488 goat anti-mouse IgG (THERMO FISHER SCIENTIFIC Invitrogen #A11034) or Alexa Fluor 594 goat anti-rabbit IgG (THERMO FISHER SCIENTIFIC Invitrogen #A11037) secondary antibody diluted 1:500 in the commercial antibody diluent for 1h at room temperature. Tissue sections were counterstained with Hoechst 33342 (10 mg/mL) (THERMO FISHER SCIENTIFIC Invitrogen #3570) diluted 1:3,000 in TBST1X for 30 minutes at room temperature before mounting the

slides in aqueous medium (FISHER SCIENTIFIC Epredia Immu-Mount #9990412) and nail polishing for securing the coverslip.

Immunostainings for DNA damage and cell cycle progression analysis were performed via incubation in Ventana buffer and staining was performed on a NEXES immunohistochemistry robot (Ventana Instruments) using an IVIEW DAB Detection Kit (Ventana) or on a Bond MAX (Leica) with antibodies against the following proteins: ɣH2AX, 1:300 dilution (Novus Biologicals); Ki67, 1:200 dilution (SP6, NeoMarkers/Lab Vision Corporation); pHH3, 1:200 (MERCK Millipore #06-570); BrdU (1:30; Roche, Switzerland).

(Immuno-)histological images were scanned using a SCN 400 slide scanner (Leica).

Image acquisition and analysis

In situ nuclear ploidy analysis of hepatocytes was performed as previously described [1] on whole-slide images (WSI) of β-catenin/Hoechst or pHH3/Hoechst-stained mouse liver tissue sections. Freshly stained slides were digitized using an Axio Scan.Z1 slide scanner coupled with a Colibri 7 multicolor LED light source fluorescent module (ZEISS) at 20x magnification (Plan-Apochromat 20x/0.8 M27 objective) and scaling of 0.325µm x 0.325µm per pixel. Fluorescence image acquisition was done with the following parameters (excitation/emission wavelengths): β-catenin (488/509 nm), pHH3 (548/561 nm) and Hoechst (353/465 nm) channels.

For cellular ploidy, mononuclear and binuclear fractions were quantified on 10 random high-power fields on scans of β-catenin/Hoechst stained liver sections. Hepatocyte nuclear ploidy was inferred from Hoechst staining and nuclear area with a specific method based on stereological image analysis and implemented in ZEN (ZEISS) and Image J software. Briefly, after Hoechst fluorescence intensity adjustment and automatic segmentation of all nuclei on the WSI, the density function of the nuclear area was plotted, and a Gaussian mixture model was fit to the multimodal distribution. Secondly, area thresholds were graphically determined to separate 2n (>30-60 µm2), 4n (>60-90 µm2) and ≥8n (>90-500 µm2) nucleus populations. The proportion of hepatocyte nuclei in each nuclear ploidy category was finally obtained by reporting the result to the total number of hepatocyte nuclei detected on the WSI. Non-hepatocyte nuclei from non-parenchymal cells (NPCs) were filtered out based on circularity (close to 1 for a hepatocyte nucleus) and nuclear area. Circularity thresholds were set at 0.8 in non-tumoral liver tissue and 0.7 in liver neoplasms to account for higher degrees of nuclear pleomorphism. Nuclei with a measured area <30 µm2 or >500 µm2 were also excluded from the analysis (NPC nuclei or incorrectly segmented nuclei with fusion or fragmentation). Three

to five liver lobes were analyzed for each mouse to account for potential inter-lobar heterogeneity. On average 80,000 ± 28,000 hepatocyte nuclei were analyzed per mouse. For nuclear ploidy map reconstruction (Figure S1), a second Image J macro was used to record the coordinates of nuclei (centroid position) which were subsequently mapped and color-coded according to their nuclear ploidy: NPC nuclei in blue, 2n hepatocyte nuclei in purple, 4n hepatocyte nuclei in green and ≥8n hepatocyte nuclei in red. To determine the proportion of G2/M hepatocyte nuclei in each nuclear ploidy category, pHH3 positivity was detected on WSI after manual thresholding. The number of positive nuclei was then reported to the total number G2/M hepatocyte nuclei per nuclear ploidy category (pHH3+ 2n, 4n and ≥8n nuclei).

Mitotic figure morphology analysis

After pHH3/Hoechst fluorescence immunostaining, liver tissue sections were analyzed directly under a Nikon Eclipse E600 upright microscope equipped with Plan Fluor 20x/0.75 and 40x/1.30 Oil objectives and 10x/22 oculars. pHH3+ hepatocyte nuclei were sought at 200x magnification, and their morphology analyzed at 400x magnification with a combination of pHH3 and Hoechst staining to better determine the mitotic stage and chromosome arrangement. G2 nuclei were discriminated from M (Mitotic) nuclei based on the nuclear morphology (presence of nuclear envelope, intermediate state of chromatin compaction and absence of chromosome individualization) and the pattern of pHH3 positivity (discrete nuclear foci overlapping heterochromatin regions). Aberrant mitotic figures (AMF) were defined as mitoses whose morphology deviates substantially from normalcy for the corresponding mitotic stage (generally bipolar and symmetrical metaphase and symmetrical anaphase with equal partitioning of chromosome)[2]. They broadly include mitotic/polar asymmetry and abnormal segregation of chromosomes in anaphase. The following AMF were considered in the present study: asymmetrical bipolar mitosis with unequal distribution of chromosomes along the metaphase plate (metaphase plate asymmetry) or unequal repartition of chromosomes in anaphase, multipolar mitosis with more than 2 spindle poles, mitosis with spindle asymmetry or abnormal spindle geometry and mitosis with abnormal chromosome segregation in anaphase (lagging chromosome and anaphase bridge). pHH3+ NPC nuclei were not considered and discriminated based on nuclear size, morphology, and localization relative to hepatocyte plates. Representative images were captured using a Nikon DS-Ri1 microscope camera and NIS-Elements Br software.

Gene Set Enrichment Analysis (GSEA)

An Agilent one-color microarray-based gene expression analysis was performed on 2 months old mice as described in [3]. Gene sets from the biological process gene ontology for GSEA analysis http://www.broadinstitute.org were downloaded from the Molecular Signatures Database or integrated manually into the GSEA. Whether genes sets were overrepresented in microarray expression data were performed with standard settings.

DNA extraction and sequencing

Genomic DNA from tumoral and non-tumoral tissues was isolated from murine liver FFPE blocks by punctions, then tissue was digested with Proteinase K overnight following manufacturer instruction (QIAamp DNA mini Kit, Qiagen). Concentration and purity of the dsDNA was determined by Qubit (Thermofisher) before sending to sequencing. Mouse whole exome capture (Agilent SureSelect Mouse All Exon Kit), library preparation and sequencing (IlluminaPE150 Q30>80%) was performed by Novogene.

WES analysis

The targeted exonic regions were sequenced to an average depth of 50. Whole exome data preprocessing, filtering and analysis were carried out according to the GATK best practices workflow for "Somatic short variant discovery (SNVs + Indels)" as described on the https://gatk.broadinstitute.org/ website. The workflow is described in detail in the supplementary methods section of [4] and was applied to the mouse whole exome data of this study using the genome reference GRCm38.p6 while Mutect2 was run in paired mode without a panel of normals (PoN). The resulting variant calls were summarized and visualized using the maftools R package [5]. The chromosomal instability score was determined using the PureCN package as described in [4]. Mutational signature analysis was conducted using the MutationalPatterns Bioconductor R package [6].

Supplemental References.

[1] Bou-Nader M, Caruso S, Donne R, Celton-Morizur S, Calderaro J, Gentric G, et al. Polyploidy spectrum: A new marker in HCC classification. Gut 2020;69:355–64. https://doi.org/10.1136/gutjnl-2018-318021.

[2] Donovan TA, Moore FM, Bertram CA, Luong R, Bolfa P, Klopfleisch R, et al. Mitotic Figures—Normal, Atypical, and Imposters: A Guide to Identification. Vet Pathol 2021;58:243–57. https://doi.org/10.1177/0300985820980049.

[3] Boege Y, Malehmir M, Healy ME, Bettermann K, Lorentzen A, Vucur M, et al. A Dual Role of Caspase-8 in Triggering and Sensing Proliferation-Associated DNA Damage, a Key Determinant of Liver Cancer Development. Cancer Cell 2017;32:342-359.e10. https://doi.org/10.1016/j.ccell.2017.08.010.

[4] Weber P, Künstner A, Hess J, Unger K, Marschner S, Idel C, et al. Therapy-Related Transcriptional Subtypes in Matched Primary and Recurrent Head and Neck Cancer. Clin Cancer Res an Off J Am Assoc Cancer Res 2022;28:1038–52. https://doi.org/10.1158/1078-0432.CCR-21-2244.

[5] Mayakonda A, Lin DC, Assenov Y, Plass C, Koeffler HP. Maftools: Efficient and comprehensive analysis of somatic variants in cancer. Genome Res 2018;28:1747–56. https://doi.org/10.1101/gr.239244.118.

[6] Manders F, Brandsma AM, de Kanter J, Verheul M, Oka R, van Roosmalen MJ, et al. MutationalPatterns: the one stop shop for the analysis of mutational processes. BMC Genomics 2022;23:1–18. https://doi.org/10.1186/s12864-022-08357-3.
