# Supplementary material for: Mcl-1 deficiency in murine livers leads to nuclear polyploidisation and mitotic errors: Implications for hepatocellular carcinoma
Source: JHEP Rep. 2023 Jul 11;5(10):100838. doi: 10.1016/j.jhepr.2023.100838 (PMC10472239; doi:10.1016/j.jhepr.2023.100838)
Supplement: Multimedia component 2 [file mmc2.docx]

**Journal of Hepatology**

**CTAT methods**

Tables for a “Complete, Transparent, Accurate and Timely account” (CTAT) are now mandatory for all revised submissions. The aim is to enhance the reproducibility of methods.

- Only include the parts relevant to your study
- Refer to the CTAT in the main text as ‘Supplementary CTAT Table’
- Do not add subheadings
- Add as many rows as needed to include all information
- Only include one item per row

**If the CTAT form is not relevant to your study, please outline the reasons why:**

|  |
| --- |

- 1. **Antibodies**

| **Name** | **Supplier** | **Cat no.** | **Clone no.** |
| --- | --- | --- | --- |
| Anti-BrdU antibody, Mouse monoclonal | Sigma-Aldrich | Cat#B8434; RRID AB476811 | BU-33 |
| Anti-β-catenin | BD BIOSCIENCES | #610154 | Monoclonal (14/β -Catenin) |
| Anti-β-tubulin | Cell Signaling Technology | #86298 | Anti-β-tubulin |
| Anti-pHH3 | MERCK Millipore | #06-570 | Polyclonal |
| Alexa Fluor 488 goat anti-mouse IgG | THERMO FISHER SCIENTIFIC Invitrogen | #A11034 | Polyclonal |
| Alexa Fluor 594 goat anti-rabbit IgG | THERMO FISHER SCIENTIFIC Invitrogen | #A11037 | Polyclonal |
| Hoechst 33342 | THERMO FISHER SCIENTIFIC Invitrogen | #3570 | N/A |
| Anti-ɣH2AX | Cell Signaling Technology | #5438S | Monoclonal |
| Anti-Ki67 | Abcam | #ab16667 | Monoclonal |

- 1. **Cell lines**

| **Name** | **Citation** | **Supplier** | **Cat no.** | **Passage no.** | **Authentication test method** |
| --- | --- | --- | --- | --- | --- |
| N/A |  |  |  |  |  |

- 1. **Organisms**

| **Name** | **Citation** | **Supplier** | **Strain** | **Sex** | **Age** | **Overall n number** |
| --- | --- | --- | --- | --- | --- | --- |
| Mcl-1^wt/wt^ | [1] | JT. Opferman | C57BL/6 | M | 2 months | 24  (this paper + [2]) |
| Mcl-1^flox/flox^ | [1] | JT. Opferman | C57BL/6 | M | 2 months | 22  (this paper + [2]) |
| Mcl-1^wt/wt^ | [1] | JT. Opferman | C57BL/6 | M/F | 12 months | 7  (this paper + [2] |
| Mcl-1^flox/flox^ | [1] | JT. Opferman | C57BL/6 | M/F | 12 months | 15  (this paper + [2]) |

- 1. **Sequence based reagents**

| **Name** | **Sequence** | **Supplier** |
| --- | --- | --- |
| N/A |  |  |

- 1. **Biological samples**

| **Description** | **Source** | **Identifier** |
| --- | --- | --- |
| Liver tissue from WT mice (2months) | This paper, [2] | N/A |
| Liver tissue from Mcl-1^Dhep^ mice (2months) | This paper, [2] | N/A |
| Liver tissue after two-third partial hepatectomy | This paper | N/A |
| Liver tissue from mice after vitamin E diet | [2] | N/A |
| Liver tissue from WT mice (12months) | This paper, [2] | N/A |
| Liver tissue from Mcl-1^Dhep^ mice (12months) | This paper, [2] | N/A |

- 1. **Deposited data**

| **Name of repository** | **Identifier** | **Link** |
| --- | --- | --- |
| Mouse RNA expression data | [2] | The accession number is [GSE75730](https://www.ncbi.nlm.nih.gov/geo/query/acc.cgi?acc=GSE75730) |
| Datasets for Gene Set Enrichment Analysis (GSEA) | Molecular signatures database (version 4.2.0) | <http://www.broadinstitute.org> |

- 1. **Software**

| **Software name** | **Manufacturer** | **Version** |
| --- | --- | --- |
| R statistical platform | R Core Team (2018) | 4.1.0 |
| Rstudio | Posit, PBC | 2022.07.2 |
| GATK | Broad Institute | 3 and 4 |
| Samtools | John Marshall and Petr Danecek et al | 1.15 |
| ZEN Blue Edition | Zeiss | v3.1 |
| Image J | NIH | v1.53 |
| NIS-Elements Br software | Nikon | v3.22.0 |

- 1. **Other (e.g. drugs, proteins, vectors etc.)**

| RNeasy Mini kit | Qiagen | cat#74106 |
| --- | --- | --- |
| Quantitect Reverese Transcription kit | Qiagen | cat#205313 |
| Taqman^TM^ Copy Number Assays | Thermo Fisher | cat#4400291 |

- 1. **Please provide the details of the corresponding methods author for the manuscript:**

| Dr. Achim Weber & Dr Laure-Alix Clerbaux  Institute of Molecular and Cancer Research (IMCR), University of Zürich (UZH), Switzerland  [achim.weber@usz.ch](mailto:achim.weber@usz.ch)  [laure-alix.clerbaux@uclouvain.be](mailto:laure-alix.clerbaux@uclouvain.be) |
| --- |

**2.0 Please confirm for randomised controlled trials all versions of the clinical protocol are included in the submission. These will be published online as supplementary information.**

| N/A |
| --- |

**References**

[1] Opferman JT, Letai A, Beard C, Sorcinelli MD, Ong CC, Korsmeyer SJ. Development and maintenance of B and T lymphocytes requires antiapoptotic MCL-1. Nature 2003;426:671–6. https://doi.org/10.1038/nature02067.

[2] Boege Y, Malehmir M, Healy ME, Bettermann K, Lorentzen A, Vucur M, et al. A Dual Role of Caspase-8 in Triggering and Sensing Proliferation-Associated DNA Damage, a Key Determinant of Liver Cancer Development. Cancer Cell 2017;32:342-359.e10. https://doi.org/10.1016/j.ccell.2017.08.010.
